# PANoptosis-based HNSCPAN-index predicts prognosis and reveals DSCAM as a therapeutic target in head and neck squamous cell carcinoma

**DOI:** 10.1371/journal.pone.0344957

**Published:** 2026-09-15

**Authors:** Yekai Feng, Yonghang Zhang, Haibo Ou, Qinglai Tang, Xiaojun Tang, Miao Zeng, Tao Chen, Yuming Zhang, Shisheng Li

**Affiliations:** 1 The 2nd Department of Head and Neck Surgery, Department of Oncoplastic Surgery, Hunan Cancer Hospital and the Affiliated Cancer Hospital of Xiangya School of Medicine, Central South University, Changsha, Hunan, China; 2 Department of Otorhinolaryngology Head and Neck Surgery, The Second Xiangya Hospital, Central South University, Changsha, Hunan, China; 3 Operation Department, The Second Xiangya Hospital, Central South University, Changsha, Hunan, China; 4 Department of Anesthesia, Hunan Cancer Hospital and the Affiliated Cancer Hospital of Xiangya School of Medicine, Central South University, Changsha, Hunan, China; Noorda College of Osteopathic Medicine, UNITED STATES OF AMERICA

## Abstract

**Objective:**

To establish a prognostic PANoptosis-related gene signature in HNSCC and elucidate the role of Down syndrome cell adhesion molecule (DSCAM) in regulating tumor progression and immune evasion.

**Methods:**

Differential expression and prognostic analyses were performed to define HNSCC subtypes based on PANoptosis-related genes. Least absolute shrinkage and selection operator (LASSO) Cox regression was applied to construct the HNSCPAN-index, which stratified patients into high- and low-risk groups. The predictive performance of this index was validated in independent cohorts. Associations with immune infiltration, mutation profiles, and drug sensitivity were also examined. Functional validation of DSCAM was conducted through in vitro and in vivo experiments.

**Results:**

Six prognostically significant genes were identified and integrated into the HNSCPAN-index, which demonstrated independent prognostic value. Immunological landscape analysis showed that the low-risk group defined by the HNSCPAN-index was enriched in CD8^+^ T cells, follicular helper T cells, and memory-activated CD4^+^ T cells. Consistently, immune, stromal, and microenvironmental scores were significantly higher in the low-risk group. DSCAM knockdown suppressed proliferation and migration of HNSCC cell lines. Knockdown of DSCAM promotes PANoptosis by inhibiting the activity of the PI3K/AKT pathway. In vivo, DSCAM depletion inhibited tumor growth and enhanced CD8^+^ T cell infiltration.

**Conclusions:**

The HNSCPAN-index may serve as a potential biomarker for prognostic stratification and prediction of therapeutic responses in HNSCC.

## Introduction

Head and neck squamous cell carcinoma (HNSCC) comprises a heterogeneous group of malignancies arising from the mucosal epithelium of the oral cavity, pharynx, and larynx [1]. Despite advances in multimodal treatment, patient outcomes remain poor due to high rates of recurrence, therapeutic resistance, and profound remodeling of the tumor microenvironment (TME) [2]. At the molecular level, HNSCC is driven by complex dysregulation of oncogenic signaling networks, including EGFR, PI3K/AKT, NOTCH1, and NF-κB pathways, which collectively promote tumor progression, immune evasion, and resistance to therapy [3]. These signaling alterations are closely intertwined with reprogramming of cell death pathways, thereby contributing to tumor survival and immune escape [3,4]. However, the mechanisms linking deregulated signaling networks to cell death regulation in HNSCC remain incompletely understood [5].

Cell death is orchestrated through multiple regulated programs, among which PANoptosis represents a distinct form of inflammatory cell death integrating pyroptosis, apoptosis, and necroptosis pathways [6]. PANoptosis can be triggered by diverse stimuli, including microbial infection, inflammatory cytokine signaling, and tumor-associated stress. Key molecular components involved in PANoptosis, such as NLRP3, caspase-1 (CASP1), GSDMD, ZBP1, CASP3, CASP8, RIPK1, and RIPK3, have been implicated in tumor suppression through coordinated regulation of inflammatory cell death [7–9]. In HNSCC, a chronic inflammation-associated malignancy, dysregulation of the NLRP3 inflammasome has been reported to promote tumor progression and enhance cancer stem cell self-renewal [10]. Emerging evidence indicates that aberrant regulation of cell death pathways significantly influences tumor behavior and therapeutic response in HNSCC [5,11]. For instance, interleukin-1β has been shown to attenuate cisplatin-induced apoptosis via caspase-3 inhibition, contributing to chemoresistance [12], whereas afatinib induces apoptosis in HNSCC cells through mTORC1 pathway suppression [13]. Collectively, these findings underscore that disruption of cell death signaling not only facilitates tumor progression and therapy resistance but also provides a rationale for developing targeted therapeutic strategies in HNSCC.

In this study, we systematically analyzed PANoptosis-related gene expression patterns in HNSCC and identified distinct molecular subtypes associated with immune infiltration and clinical outcomes. Based on these findings, we developed a PANoptosis-based prognostic model (HNSCPAN-index) for risk stratification and therapeutic response prediction. Furthermore, we identified Down syndrome cell adhesion molecule (DSCAM) as a key regulatory gene within this signature and investigated its functional role in HNSCC through integrated bioinformatic analysis and experimental validation in vitro and in vivo, aiming to elucidate its role in tumor progression, PANoptosis regulation, and immune modulation.

## Materials and methods

### Data collection and processing

RNA-Seq data for 44 non-cancerous samples, 522 HNSCC samples, and 528 HNSCC clinical data were downloaded from TCGA database (https://www.cancer.gov/tcga) [14]. The clinical data included variables such as age, sex, TNM stage, and survival outcome. Somatic mutation data were retrieved separately from TCGA. Ensembl IDs were translated into an expression matrix profile using Perl, enabling the conversion of these identifiers into gene symbols. The validation dataset (GSE65858, n = 270) was acquired from the GEO database (https://www.ncbi.nlm.nih.gov/geo/) [15]. The PANoptosis gene list was compiled from the literature (S1 Table) [16,17].

### Ethics statement

TCGA and GEO belong to public databases. The patients involved in the database have obtained ethical approval. Users can download relevant data for free for research and publish relevant articles. The animal study procedures were reviewed and approved by the Ethics Committee of the Second Xiangya Hospital, Central South University (approval no. 20250906), and were conducted in accordance with its guidelines.

### Differential expression and survival analysis of PANoptosis genes in HNSCC

To evaluate the differentially expressed of PANoptosis genes between normal and HNSCC tissues, we conducted two sets of t-tests on the log2-transformed expression data. Box plots were generated using the “ggpubr” package. Overall survival (OS) was selected as the endpoint to elucidate the correlation between PANoptosis genes and prognostic outcomes.

### Consensus clustering of PANoptosis in molecular subtypes of HNSCC

To elucidate the characteristics of PANoptosis genes, we employed the “ConsensusClusterPlus” R package to stratify patients with HNSCC into three distinct clusters. Kaplan-Meier method was used to assess OS across three distinct clusters. Additionally, single-sample Gene Set Enrichment Analysis (ssGSEA) method was performed to compare immune cell infiltration in different clusters to understand differences in the immune microenvironment of different molecular subtypes [18].

### Identification of HNSCC differential genes associated with PANoptosis

Gene Set Variation Analysis (GSVA) was performed using gene expression profiles and phenotype classifications to assess the pertinent pathways and underlying molecular mechanisms [19]. Criteria for identifying significantly enriched pathways between the three distinct PANoptosis clusters included | logFC| > 0.1 and adj. *p* < 0.05. Using the “limma” package, we performed differential expression gene analysis between PANoptosis molecular subtypes, with the criteria set at |logFC| > 0.585 and adj. *p* < 0.05 to identify significantly changed genes. The “clusterProfiler” package is employed to conduct the KEGG and GO enrichment analyses of DEGs [20–23].

### Survival analysis and machine learning

To identify the prognostically relevant DEGs in the TCGA-HNSCC dataset, univariate Cox regression analyses were conducted, with DEGs serving as the independent variables. The entire dataset was randomly split into training and validation cohorts in an approximate 1:1 ratio. To refine the number of prognostically relevant DEGs, LASSO Cox regression analyses were conducted using the R “glmnet” package. The final prognostic risk score was subsequently constructed through multivariate Cox regression. The formula for the prognostic risk score is presented below:


$$\begin{array}{c} Risk\;score\;=\;\sum_{i=\text{1}}^{n}bi*\;Ei, \end{array}$$

where *n* denotes the number of DEGs associated with prognosis within the risk model, *b* represents the regression coefficients for multivariate Cox regression analysis, and *E* indicates the expression levels of the risk-related DEGs. Risk difference analysis was performed to evaluate variations in risk scores across subgroups. Utilizing data from the TCGA and GEO databases, we classified patients into high-risk and low-risk groups based on the 50th percentile of their risk coefficients. Prognostic differences were analyzed using the R “survival” package, with long-rank tests determining statistical significance. Additionally, we developed a nomogram to predict OS for patients with HNSCC by integrating risk scores and clinical data, utilizing the R packages “survival” and “rms”.

### Predicting the immune landscape, immunotherapy response, and drug responses

The CIBERSORT algorithm was applied to estimate the relative proportions of immune cell subsets in the TCGA-HNSCC dataset by quantifying the relative abundance of RNA transcripts specific to various immune cell types [24]. Immuno-score, Stromal-score, and ESTIMATE score were computed using the ESTIMATE algorithm. TIDE score was used to predict tumor response to immune checkpoint inhibitors. Somatic mutations were evaluated utilizing the “maftools” R package. The TMB grade was estimated by dividing the number of non-synonymous mutations through the total genome size in megabases. Additionally, the therapeutic drugs’ IC50 was predicted using the “prophytic” R package. Data on immunotherapy cohorts are available from the public database IMvigor210 [25].

## Validation and functional characterization of the HNSCPAN-index

### Cell culture

HEK293T, MOC1, CAL-27 and HN30 cells were cultured in a complete DMEM medium supplemented with 10% fetal bovine serum (Gibco), while Human bronchial epithelial (16HBE) cells were cultured in complete RPMI-1640 medium. All cell lines were purchased from the Cell Bank of the Type Culture Collection of Chinese Academy of Sciences (Shanghai, China). HNSCC cell lines were treated with LPS and ATP to induce pyroptosis, following the protocol outlined in Ref [26].

### Lentiviral transduction and RNA interference

Lentivirus was produced by co-transfecting HEK293T cells with the plasmid of target gene and lentiviral packaging plasmids (Addgene) using TurboFect (Thermo Fisher Scientific, R0531) according to the manufacturer’s instructions. The viral supernatants were then collected and used to transduce target cells in the presence of polybrene (Sigma-Aldrich, H9268). After 24 hours, the medium was replaced. Puromycin (Thermo Fisher Scientific, A1113803) selection (2 μg/mL) was initiated 48 hours post-transduction and maintained until all non-transduced control cells were eliminated. Knockdown efficiency was quantified by Western blotting. Transfection for CAL-27 and HN30 cells was performed using Lipofectamine® 3000 (Invitrogen). Small interfering RNA (siRNA) was utilized to knock down the expression of the target gene.

The sgRNA sequence of DSCAM was 5’-CACCGATTCGCGAAGCTCTGGAACA-3’. The siRNA sequence of DSCAM were as follows: si-1 5’-CGUGUUGGUUCAACCACAATT-3’, si-2 5’-GGACAAGCUGUCCGCUAATT-3’.

### qRT-PCR

Using a 6-well plate, cells are washed twice with PBS and treated with 1 mL Trizol reagent (Invitrogen) per well. After pipetting and incubation, chloroform is added, shaken, and centrifuged to separate phases. The upper phase is collected, mixed with isopropanol to precipitate RNA, which is then washed with 75% ethanol, air-dried, and dissolved in nuclease-free water for quality assessment. Reverse transcription kit (Yeasen, Shanghai, China) is performed to generate cDNA, with GAPDH as the internal control for Real-time PCR, run in triplicate. Gene expression is analyzed via the 2^-△△Ct^ method. Primer sequences are listed in S2 Table.

### Western blotting

Western blotting was performed as previously standard techniques [27]. Cells were washed with phosphate-buffered saline (PBS) and lysed on ice for 30 minutes in RIPA buffer supplemented with protease and phosphatase inhibitors. Following sonication and centrifugation, protein concentration was quantified using the BCA assay (Elabscience, Wuhan, China) at 595 nm. Proteins were denatured in 5 × loading buffer at 95°C for 10 minutes and then separated by SDS-PAGE. After transferring the proteins to a PVDF membrane at 300 mA for 90 minutes, the membranes were blocked with 5% skim milk and incubated overnight at 4°C with the primary antibody. The membranes were then incubated with a secondary antibody for one hour at room temperature. Detection was performed using enhanced chemiluminescence (ECL), and images were captured for further analysis. Antibody information is listed in S3 Table.

### Flow cytometry

After harvesting and filtering through a 200-mesh strainer, cells were washed and resuspended in staining buffer (PBS containing 1% BSA). Cell concentration was adjusted to 1 × 10^7^ cells/mL. Aliquots of 1 × 10^6^ cells per tube were incubated with Fc receptor blocking reagent, followed by staining with cell surface markers using fluorochrome-conjugated antibodies for 30 min at 4°C in the dark. For intracellular staining, cells were subsequently fixed and permeabilized using Intracellular Fixation/Permeabilization Buffer Kit (Elascience, Wuhan, China) according to the manufacturer’s instructions, followed by incubation with antibodies against intracellular targets. Appropriate controls, including unstained, isotype, and single-color compensations, were set up. After staining, cells were washed, resuspended in 200 μL of staining buffer, and analyzed. Antibody information is listed in S4 Table.

### *In vitro* experiments

After treating cells in a 96-well plate, a working solution was prepared by mixing CCK8 reagent (Elabscience, Wuhan, China) with complete medium at a 9:1 ratio, using 10 µL of CCK8 per well. Following the removal of the old medium, 100 µL of the CCK8 solution was added to each well, including control wells without cells, and incubated at 37°C in the dark for 2 hours. Absorbance at 450 nm was measured using a microplate reader, and cell viability was calculated according to the appropriate formula, with at least three replicates per group.

Cell migration was evaluated using a wound-healing assay. HNSCC cells were seeded in 6-well plates. After the transfection procedure was accomplished, 200 µL pipette tip was employed to create a scratch in the center of each well. PBS was utilized for washing to remove the detached cells, and the remaining cells were cultivated under starvation conditions. Gap distances at 0, 24 and 36 hours were recorded using an inverted microscope.

Cell invasion was further investigated using a transwell assay. First, thaw the ECM gel (Invitrogen) and dilute it with serum-free medium at a 1:8 ratio. Add the diluted solution to the upper chamber of the Transwell and incubate overnight at 37 °C for gelation. Next, prepare a serum-free cell suspension, introduce approximately 2000 cells into the upper chamber, and add 500 µL of 10% FBS medium to the lower chamber. Finally, after 36 hours, wash the upper chamber, fix and stain the cells, and assess invasion capacity by counting them under a microscope.

### *In vivo* experiments

For the subcutaneous tumor models, tumor cells were harvested, resuspended in serum-free basal medium, and subcutaneously inoculated into the right flank of mice (5 × 10^6 cells in 100 μL per mouse). Tumor dimensions and body weights were measured every two days. Tumor volume was calculated using the formula: Volume = (Length × Width^2^)/2. All mice were humanely euthanized when the tumor diameter approached 2 cm, in accordance with institutional animal ethics guidelines.

### Immunofluorescence

Fresh tissues were fixed, paraffin-embedded, and sectioned (35 μm). Following deparaffinization and rehydration, two rounds of high-pressure antigen retrieval were performed. After blocking endogenous peroxidase and non-specific binding, sections were sequentially incubated with the first primary antibody, HRP-conjugated secondary antibody, and TSA reagent. After the second antigen retrieval, the second primary antibody, HRP-secondary antibody, and TSA were applied. Nuclei were stained with DAPI, and images were acquired by fluorescence microscopy.

### Statistical analysis

Data analysis in this study utilized R software (version 4.2.1) and GraphPad Prism software (version 9.4.1). The Wilcoxon rank-sum test assessed mean differences between two groups, while the Kruskal-Wallis test evaluated differences among three or more groups. For survival analysis, the Kaplan-Meier method generated survival curves, with the log-rank test used to assess the significance of differences between these curves.

## Results

### Identification of potential PANoptosis-related subtypes in TCGA-HNSCC cohort

**Fig 1:**
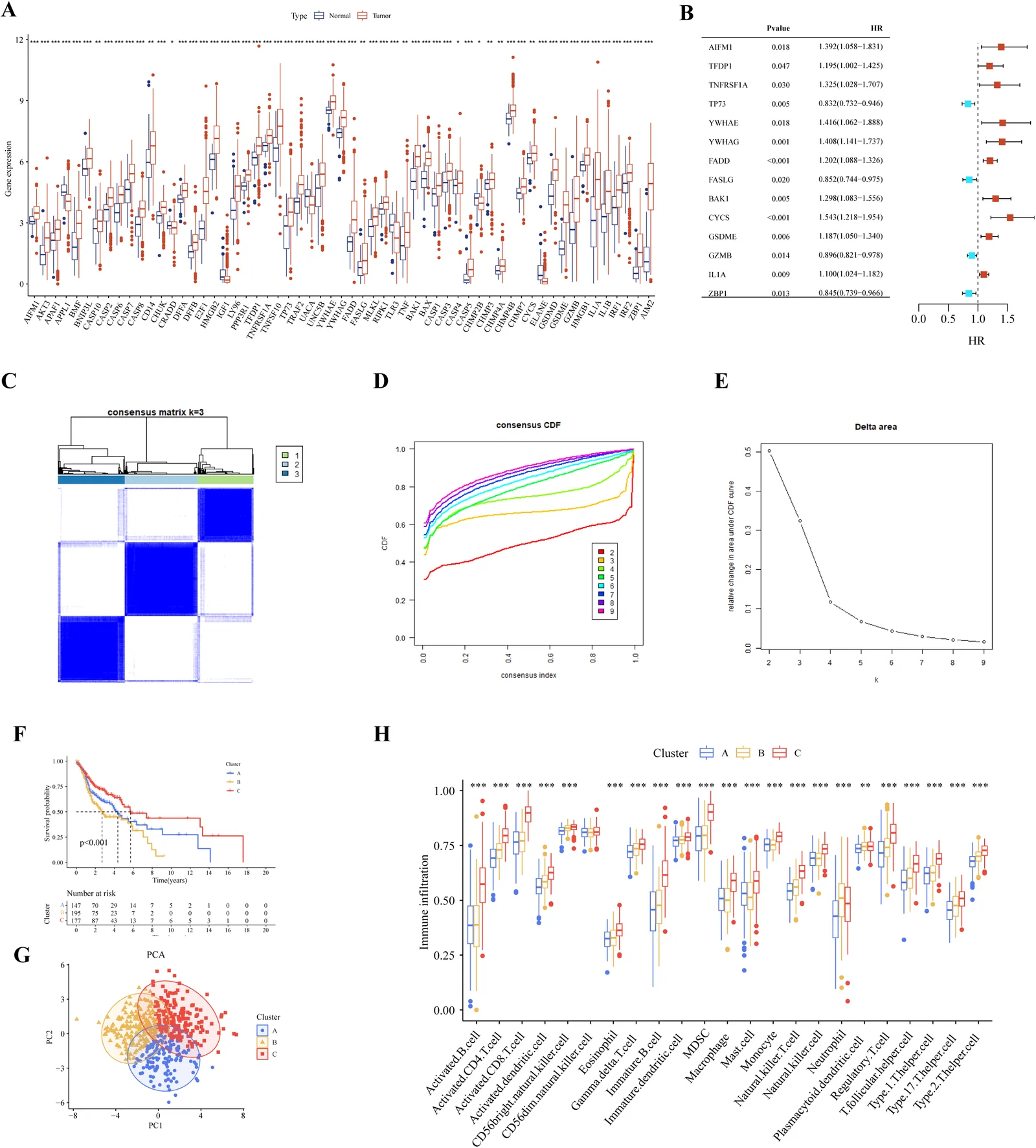
Identification of potential PANoptosis-related subtypes in TCGA-HNSCC cohort. (**A**) Differential mRNA expression of PANoptosis genes in tumors and adjacent non-cancerous tissues. (**B**) Forest plot lists the PANoptosis genes associated with the prognosis of HNSCC. (**C**) PANoptosis Clusters were identified using consensus clustering. (**D**, **E**) When k = 2 to 9, the average consistency within clusters and the area under the CDF curve. (**F**) Significant differences in OS between the three PANoptosis clusters. (**G**) PCA delineated transcriptional architecture across the three clusters. (**H**) Box plot showing the distinct patterns of immune cell infiltration across the three identified PANoptosis clusters. * *p* < 0.05, ** *p* < 0.01, and *** *p* < 0.001.

The PANoptosis gene set comprised 28 pyroptosis genes, 32 apoptosis genes, and 8 necrosis genes. We explored the differential expression of these 68 genes in samples from 522 patients with HNSCC. Among them, 59 genes (86.7%) exhibited differential expression between normal and tumor tissues (Fig 1A). Furthermore, univariate Cox regression analysis identified 14 genes (Fig 1B), accounting for 20.5% of the total, that are significantly related with patient prognosis. Elevated expression levels of these genes appear to be correlated with poor clinical outcomes. Based on the expression profiles of PANoptosis genes, the cohort of 520 HNSCC samples was stratified into three distinct molecular clusters: A, B, and C, consisting of 147, 195, and 178 patients, respectively. The optimum number of clusters was determined to be K = 3 (Fig 1C-E). To assess the reliability of the molecular subtypes, a KM survival analysis was performed, which showed significant differences in OS across subtypes. Notably, Cluster B exhibited markedly poorer OS outcomes than those of clusters A and C. (*p* < 0.05, Fig 1F). PCA analyses of the HNSCC population confirmed that patients with the three subtypes were unevenly distributed in the TCGA dataset (Fig 1G). Box plot, generated using the ssGSEA algorithm, demonstrated the distribution of immune cell infiltration across the three clusters. Cluster C exhibited the highest degree of immune cell infiltration, correlating with a more favorable prognosis. Conversely, Clusters A and B that demonstrated poorer OS were characterized by lower levels of immune infiltration. In addition to antitumor immune cells, immunosuppression-associated myeloid-derived suppressor cells and CD8^+^ T cells significantly infiltrated Cluster C (Fig 1H). The enhanced prognosis observed in HNSCC tumors with high CD8^+^ T cell infiltration may be attributed to the augmented effector function of these cells and their increased capacity for tumor cell eradication [28]. Differentially expressed genes identified by single-cell sequencing of HNSCC samples have been linked to distinct tumor subtypes [29]. Notably, certain genes such as the PANoptosis-related gene TNF were associated with immune activation and may exhibit context-specific responses within various TMEs. This observation implies that the degree of immune cell infiltration may reflect the expression levels of PANoptosis genes.

### Functional analysis of differentially expressed genes associated with PANoptosis in HNSCC

**Fig 2:**
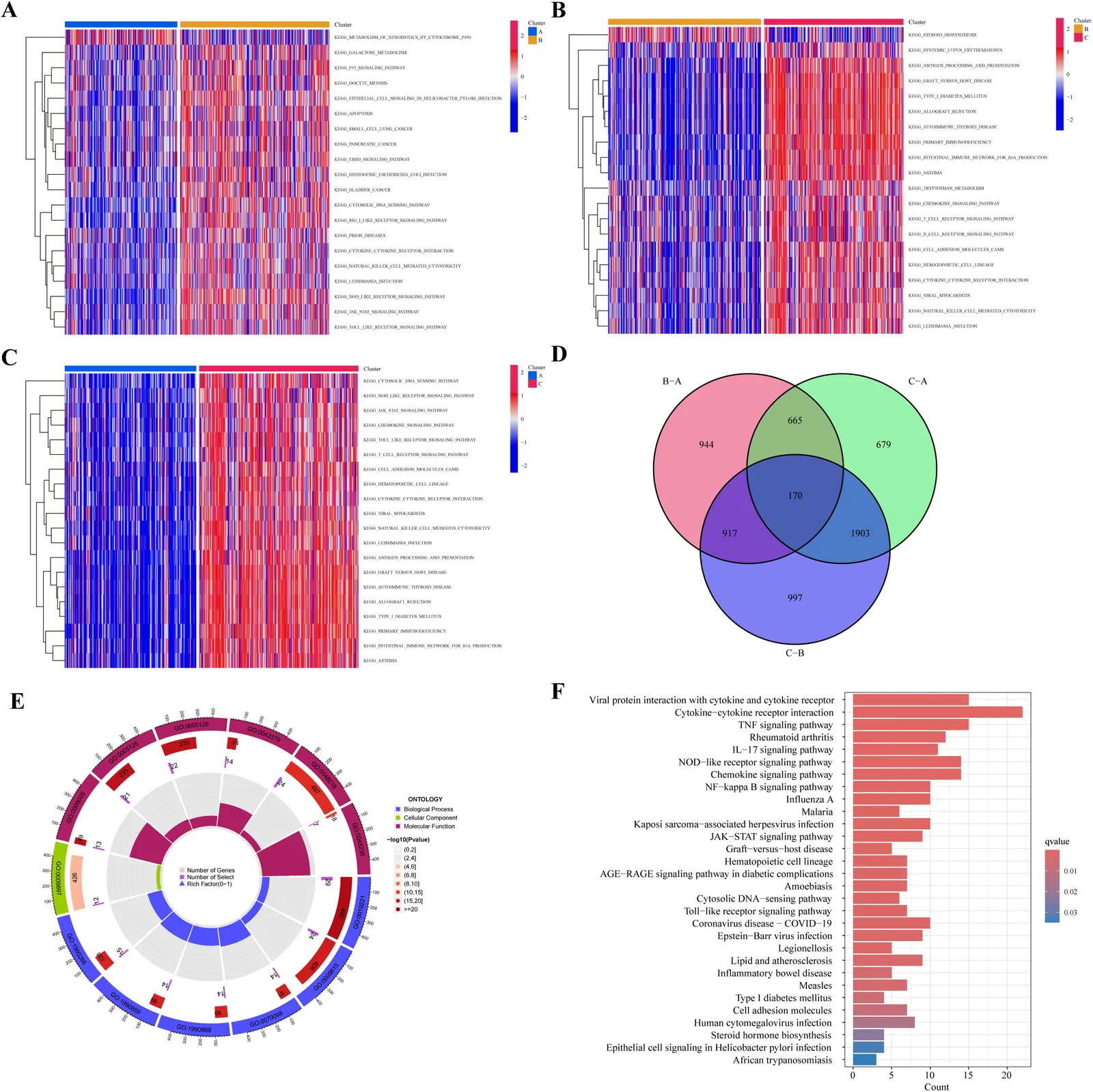
Functional enrichment analysis of HNSCPAN_DEGs. (**A-C**) GSVA was used to compare the differences in pathway activity between the following pairs: Clusters A and B (**A**), Clusters B and C (**B**) Clusters A and C (**C**). (**D**)The Venn diagram showing differentially expressed genes crossed between three Clusters. (**E**) The results of GO enrichment analyses based on the HNSCPAN_DEGs. (**F**) The results of KEGG enrichment analyses based on the HNSCPAN_DEGs.

GSVA revealed significant pathway activity disparities among clusters A, B, and C, highlighting distinct biological pathway enrichment patterns across these molecular subgroups. For example, Cluster C demonstrated significantly higher enrichment in pathways related to immune function compared to Clusters A and B. These pathways included the T cell receptor signaling pathway, primary immunodeficiency, antigen processing and presentation, the intestinal immune network for IgA production, natural killer cell-mediated cytotoxicity, and the NOD-like receptor signaling pathway. In contrast, Cluster B exhibited greater enrichment in RIG-I-like receptor signaling and apoptosis pathways than Cluster A (Fig 2A–C). Mapping the gene sets across the three clusters identified 170 DEGs associated with PANoptosis in HNSCC (HNSCPAN_DEGs) (Fig 2D). Enrichment analysis of these DEGs revealed their involvement in immune system processes, inflammatory responses, and cell signaling. The principal biological process enriched among these DEGs was the cytokine-mediated signaling pathway, with chemokine-mediated signaling also playing a prominent role. In terms of cellular composition, DEGs were primarily enriched on the external side of the plasma membrane. Molecular functions analysis showed substantial enrichment in chemokine activity and receptor-ligand interactions. KEGG pathway enrichment analysis further demonstrated that HNSCPAN_DEGs were predominantly enriched in pathways such as NOD-like receptor, cytokine–cytokine receptor interactions, JAK-STAT, TNF, and the AGE-RAGE signaling pathway in diabetic complications (Fig 2E, F).

### Construction and validation of the HNSCPAN-index prognostic model

**Fig 3:**
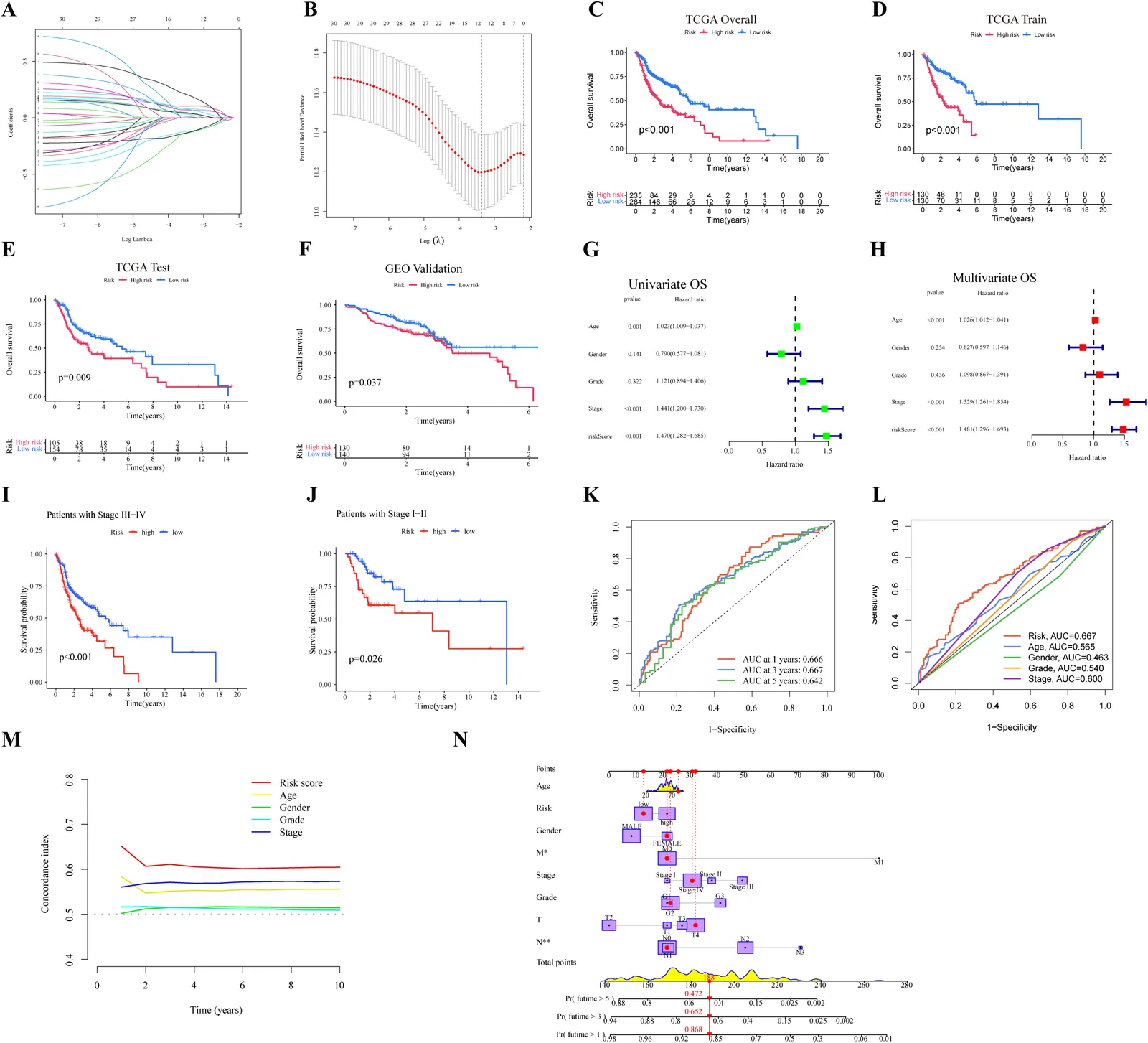
Establishment and validation of the HNSCPAN-index prognostic model. (**A**) Coefficient trajectories of prognostically relevant differentially expressed genes. (**B**) LASSO Cox regression analysis conducted based on the minimum criterion. (**C-F**) KM survival curves illustrate the prognostic relevance of the HNSCPAN-index across the TCGA overall cohort, as well as in the training, test, and GEO validation datasets. (**G**, **H**) Univariate and multivariate Cox regression demonstrates HNSCPAN-index as an independent risk factor. (**I**, **J**) Kaplan-Meier analysis revealed significant survival differences between high- and low-risk groups in stages I-II and III-IV. (**K**) HNSCPAN-index demonstrated predictive AUC values for 1-, 3-, and 5-year overall survival in patients. (**L**) The AUC of HNSCPAN-index for the 3-year OS was greater than those of other prognostic factors. (**M**) The predictive accuracy of the risk score, assessed by the C-index, outperforms that of other prognostic indicators. (**N**) A nomogram for predicting prognosis of HNSCC patients.

To assess the effect of HNSCPAN_DEGs on survival outcomes, six gene features that exhibited a robust correlation with prognosis were identified by LASSO and univariate Cox regression analyses. These findings culminated in the construction of a PANoptosis risk score model based on HNSCPAN_DEGs, designated as the HNSCPAN-index (Fig 3A, B). The HNSCPAN-index was calculated as follows: HNSCPAN-index = 0.4198 * DSCAM + 0.1734 * NT5E + 0.1147 * CXCL1–0.3343 * MIAT – 0.2405 * IL12RB2–0.1253 * AKR1C3. Using the median risk score, patients with HNSCC were stratified into low-risk and high-risk cohorts. To validate the predictive accuracy of these prognostic characteristics, we randomly assigned 519 patients with complete survival data into training and test groups. Within the training set (n = 260), patients were categorized into a high-risk group (n = 130) and a low-risk group (n = 130) based on the HNSCPAN-index. OS was significantly better in the low-risk group than in the high-risk group. Similar outcomes were observed in the TCGA test (n = 259) and GEO validation sets (GSE65858, n = 270) (Fig 3C-F). The independent prognostic value of the HNSCPAN-index was further confirmed via both univariate and multivariate Cox regression analyses (Fig 3G, H). Analysis of TNM staging revealed that patients classified as stage I and II were predominantly found in the low-risk group, while stages III and IV were primarily enriched in the high-risk group (Fig 3I, J). ROC curve analysis was employed to validate the model’s specificity and sensitivity, yielding area under the curve (AUC) values for the HNSCPAN-index of 0.666, 0.667, and 0.642 for 1-, 3-, and 5-year overall survival, respectively, surpassing those of other factors (Fig 3K, L). Moreover, the concordance index (C-index) indicated that the HNSCPAN-index exhibited superior predictive power compared to other clinical characteristics (Fig 3M). We also constructed a nomogram integrating the risk scores with clinical factors, enhancing its applicability for predicting patient survival in clinical settings (Fig 3N).

### Analysis of immune infiltration, mutational profiles, and DNA methylation

**Fig 4:**
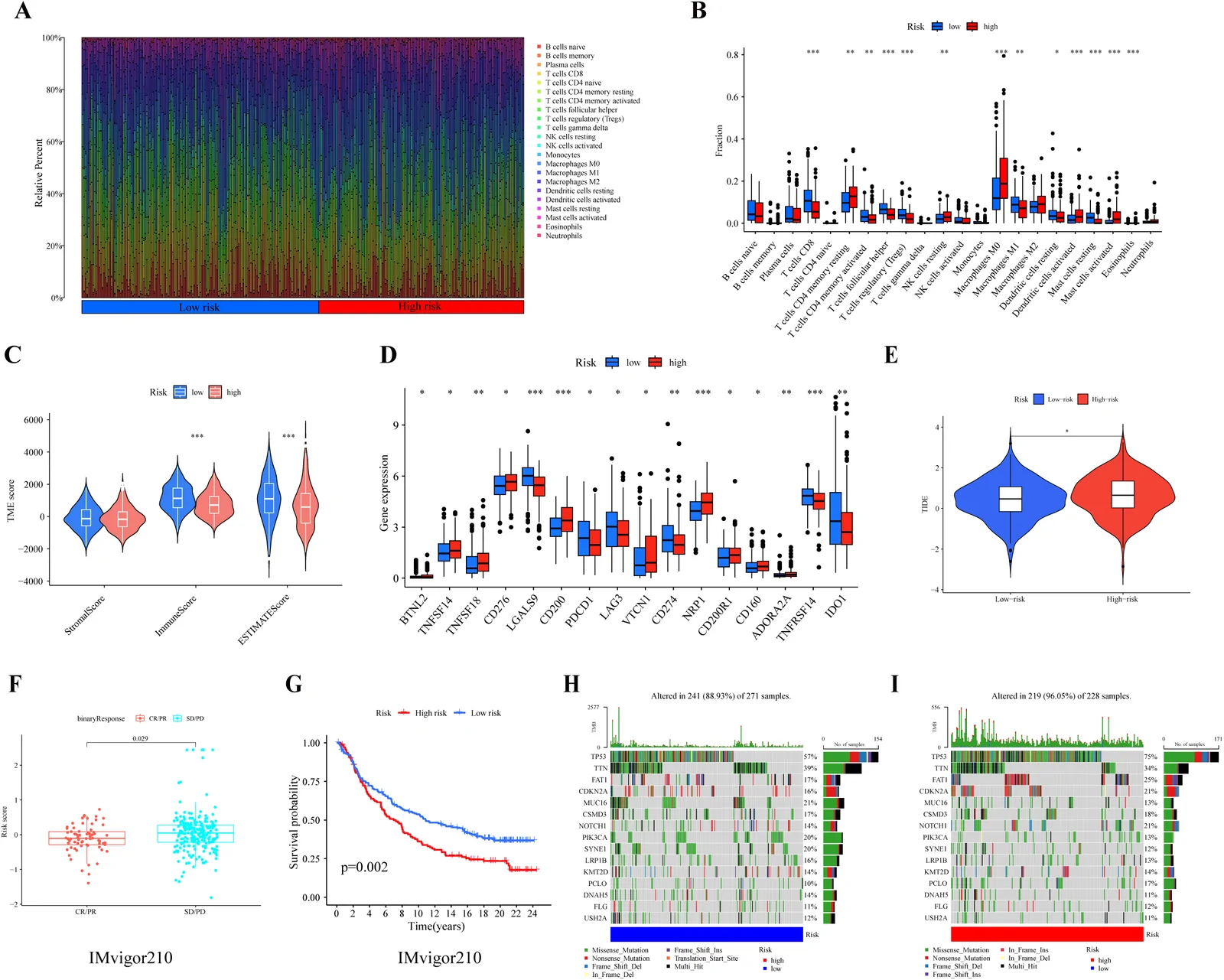
The HNSCPAN-index serves as a reliable predictor of the immune landscape, mutational landscape, and immunotherapy efficacy. (**A**) The CIBERSORT algorithm quantified 22 types of tumor-infiltrating immune cells in two risk groups. (**B**) The proportion of different immune cell infiltrations. (**C**) Stromal-score, Immuno-score and Microenvironment-score in two groups. (**D**) Differences in immune checkpoint gene expression across different groups. (**E**) Comparison of TIDE scores between the two groups. (**F**) Correlation of risk scores with CR/PR and SD/PD was explored in the IMvigor210 cohort. (**G**) Comparison of OS between two risk groups in the IMvigor210 cohort. (**H**, **I**) Waterfall plots illustrated the somatic mutation profiles of both risk groups.

Previous studies have indicated that PANoptosis modulates immune infiltration and tumor mutation rates [30,31]. To elucidate the immunological characteristics of the two risk groups defined by the HNSCPAN-index, we conducted immunological landscape analysis using the CIBERSORT and ESTIMATE algorithms. As illustrated in Fig 4A, the waterfall plot delineates the distribution of 22 immune cell types. Notably, we identified an upregulation of M0 and M2 macrophages, accompanied by a downregulation of M1 macrophages, CD8^+^ T cells, follicular helper T cells, and memory-activated CD4^+^ T cells in patients classified as high-risk (Fig 4B). Subsequently, we evaluated Immune-Score, Stromal-Score, and Microenvironment-Score between the two groups. Our findings demonstrated substantial differences in both immune and Microenvironmental scores, with the low-risk group exhibiting higher scores than the high-risk group, while stromal scores showed no significant changes (Fig 4C). These observations suggest that the high-risk group exhibits more susceptibility to immunosuppressive microenvironments.

Immune checkpoint inhibitors (ICIs) exert their anti-tumour effects by leveraging the patient’s immune system to suppress tumour growth [32]. In our analysis of immune checkpoint gene expression profiles between the two risk groups, we identified 16 genes exhibiting significant differential expression. Among these, BTNL2, TNFSF14, TNFSF18, CD276, CD200, VTCN1, NRP1, CD200R1, CD160, and ADORA2A were upregulated in the high-risk cohort. Conversely, LGALS9, PD-1 (PDCD1), LAG3, PD-L1 (CD274), TNFRSF14, and IDO1 levels were elevated in the low-risk group (Fig 4D). Patients in the high-risk group exhibited elevated TIDE scores, suggesting an increased propensity for immune evasion and a diminished response to immune checkpoint therapies compared to those in the low-risk group (Fig 4E). Further analysis of the IMvigor210 cohort corroborated these findings, revealing that the risk scores were significantly lower in the immunotherapy responder group (CR/PR) than non-responder group (SD/PD) (Fig 4F). Following immunotherapy, OS was markedly improved in the low-risk group relative to the high-risk group (*p* = 0.002, Fig 4G), suggesting that patients with lower risk scores demonstrated a favorable response to immunotherapy.

We subsequently elucidated the somatic mutation landscape across high-risk and low-risk groups. The 15 most frequently mutated genes included TP53, MUC16, TTN, FAT1, SYNE1, KMT2D, CDKN2A, CSMD3, NOTCH1, PIK3CA, USH2A, KMT2D, PCLO, DNAH5, FLG, and LRP1B. These mutations play a crucial role in cancer development and progression, particularly mutations in TP53, which exhibited a mutation rate of 75% in the high-risk group compared to 57% in the low-risk group (Fig 4H, I). This significant disparity underscores the differences in mutation frequency between the two groups and offers vital insights into the biological characteristics of the tumors. Specifically, TP53 mutations, a key tumor suppressor gene, are closely linked to the malignancy of various cancers and patient prognosis, suggesting that individuals in the high-risk group may experience more severe disease progression [33].

To further complement the immune and mutational analyses, we investigated the epigenetic regulation of the six key genes constituting the HNSCPAN-index (DSCAM, NT5E, CXCL1, MIAT, IL12RB2, and AKR1C3) using TCGA-HNSC 450K methylation array data [34]. Correlation analysis between promoter methylation and mRNA expression revealed distinct patterns: methylation of NT5E and IL12RB2 showed a clear negative correlation with gene expression, whereas DSCAM exhibited a positive correlation (S1 Fig). These findings suggest that aberrant DNA methylation may contribute to the transcriptional regulation of PANoptosis-related genes, further influencing risk stratification and tumor biology in HNSCC.

### HNSCPAN-index predicts HNSCC drug sensitivity

**Fig 5:**
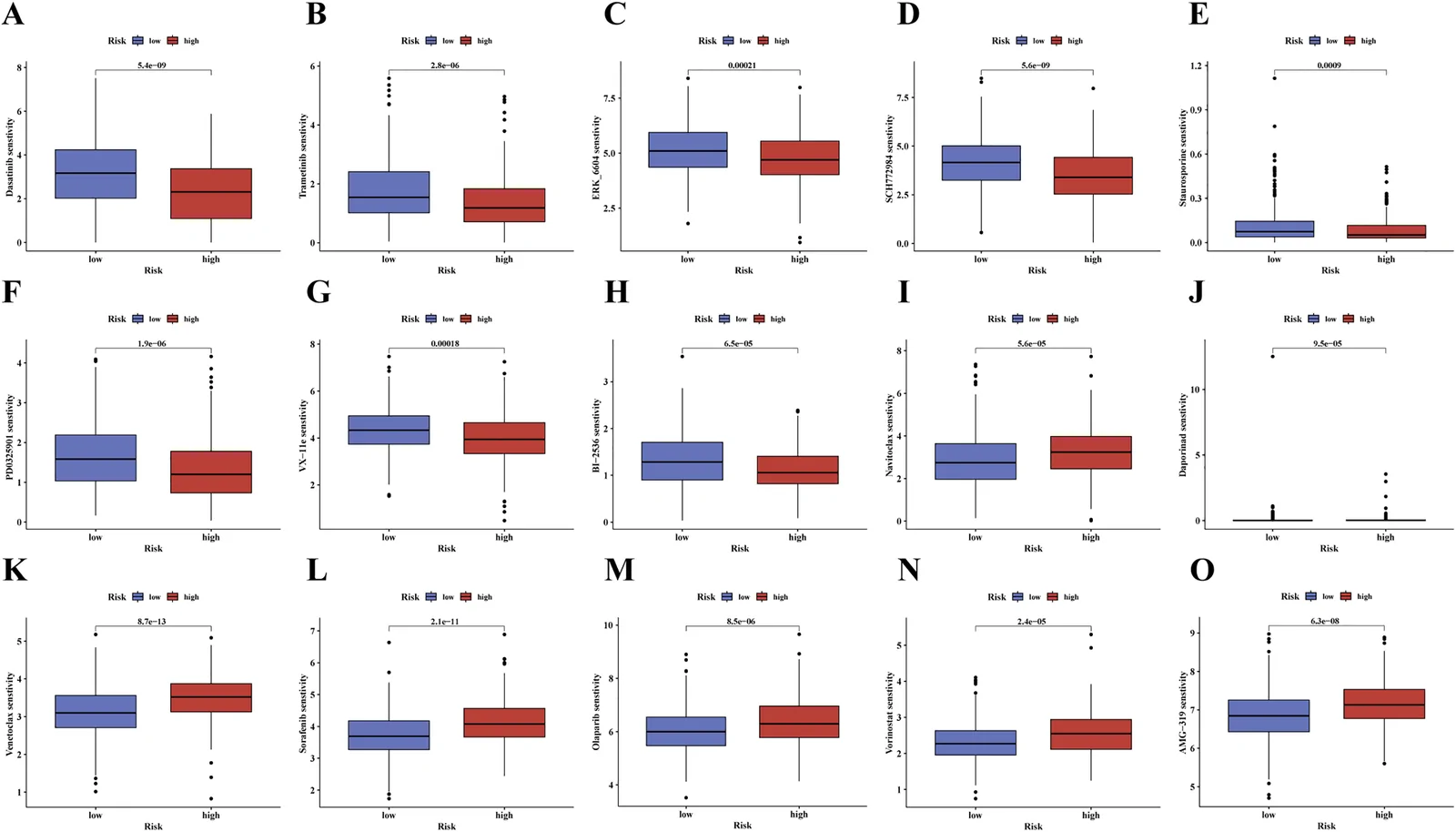
HNSCPAN-index predicts the relevance of drug sensitivity. (**A–H**) Therapy drugs to which the high-risk group is particularly sensitive compared to the low-risk group. (**I–O**) Therapeutic agents to which the low-risk group is sensitive.

ICIs have demonstrated promising clinical outcomes in patients with HNSCC and elevated survival rates among treatment-responsive individuals [35]. However, the persistence of multidrug resistance is a primary impediment to ICI efficacy. Nonetheless, combination therapies, including chemotherapy, continue to serve as the cornerstones of treatment. This study aimed to assess the IC50 values for predicting the sensitivity of distinct HNSCC populations to various therapeutic agents. Our findings revealed that the high-risk group may show predicted sensitivity to multiple agents, including Dasatinib, Trametinib, Staurosporine, ERK_6604, SCH772984, PD0325901, VX-11e, and BI-2536 (*p* < 0.001, Fig 5A–H). Conversely, the low-risk group may show predicted sensitivity to Navitoclax, Daporinad, AMG-319, Vorinostat, Olaparib, Venetoclax, and Sorafenib (*p* < 0.001, Fig 5I–O). It should be emphasized that these results still require further validation.

### Identification of key molecule DSCAM on HNSCC

**Fig 6:**
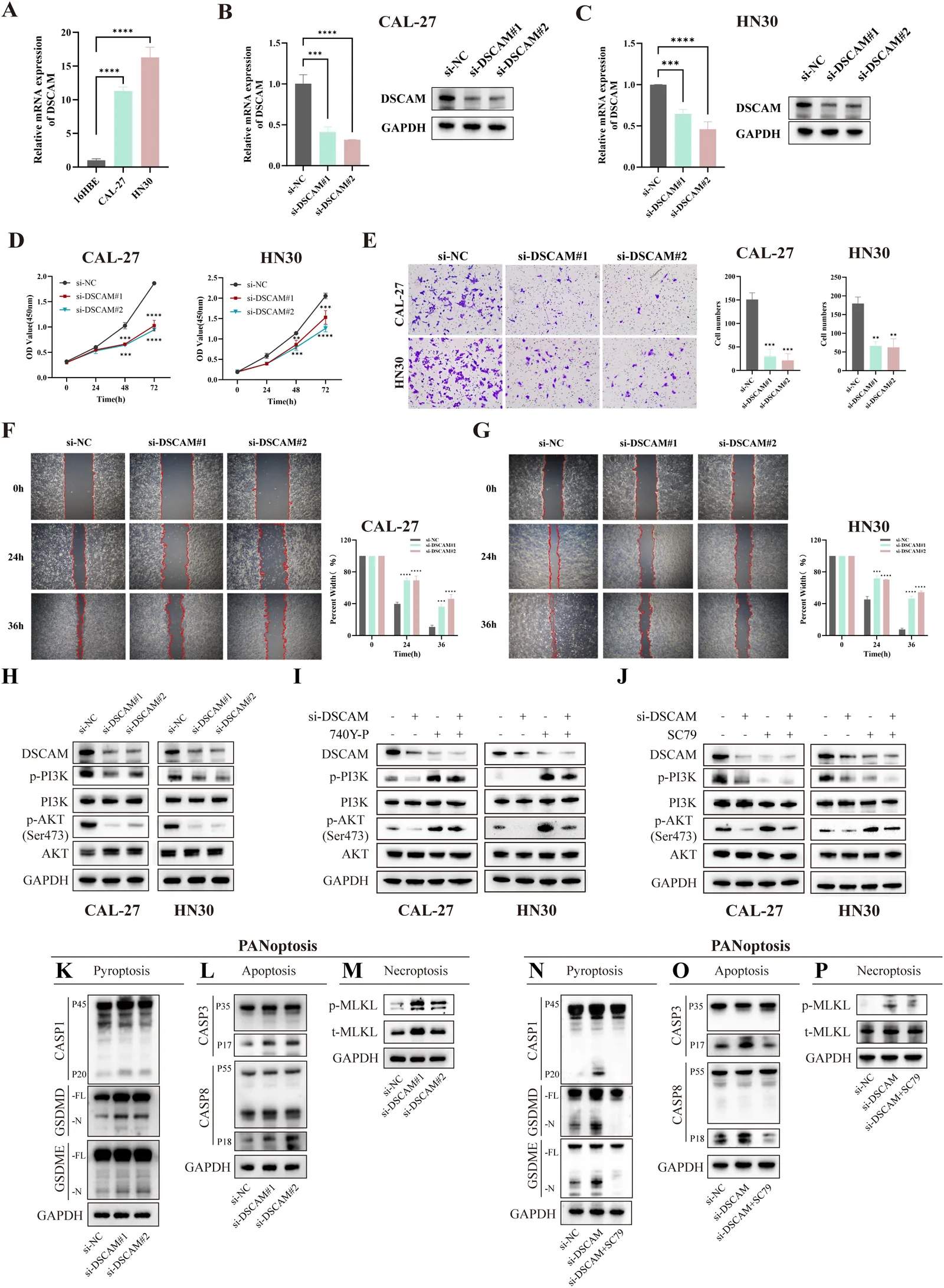
DSCAM knockdown inhibits the proliferation and migration and promotes PANoptosis of HNSCC. (**A**) RT-qPCR analysis of DSCAM mRNA was conducted in human bronchial epithelial cells (16HBE) and HNSCC cell lines (CAL-27, HN30). (**B**, **C**) qPCR and Western blot analyses confirming the transfection efficiency of si-DSCAM#1 and si-DSCAM#2. (**D**) CCK-8 assay evaluating the impact of si-DSCAM on the proliferation of CAL-27 and HN30 cells. (**E**) Transwell migration assays of CAL-27 and HN30 cells following transfection. Representative images (upper, × 200) and quantification of migrated cells (lower) are shown. (**F**, **G**) Following transfection, the migratory capacity of different groups of CAL-27 and HN30 cells was evaluated using a wound healing assay (0, 24, and 36 hours). (**H**) Western blot analysis of p-PI3K and p-AKT expression in CAL-27 and HN30 cells following DSCAM knockdown. (**I**) Protein levels of p-PI3K and p-AKT in control and DSCAM-knockdown CAL-27 and HN30 cells after treatment with 740 Y-P(50 μg/ml, 2 h). (**J**) Protein levels of p-PI3K and p-AKT in control and DSCAM-knockdown CAL-27 and HN30 cells after treatment with SC79 (10 μM, 2 h). (**K-M**) Western blot analysis of activated CASP1, cleaved GSDMD and cleaved GSDME (**K**), cleaved CASP3 and cleaved CASP8 (**L**), and p-MLKL and total MLKL (**M**) in CAL-27 cells transfected with si-NC, si-DSCAM #1, or si-DSCAM #2. GAPDH served as the loading control. (**N-P**). Western blot analysis of activated CASP1, cleaved GSDMD and cleaved GSDME (N), cleaved CASP3 and cleaved CASP8 (O), and p-MLKL and total MLKL (P) in CAL-27 cells transfected with si-NC, si-DSCAM, or si-DSCAM combined with SC79. Data are expressed as mean ± SD, n = 3. *p < 0.05, **p < 0.01, ***p < 0.001.

Given that genes in the HNSCPAN-index such as NT5E, MIAT, and CXCL1 have been previously validated in HNSC [36–38], while the DSCAM gene, which has the highest correlation coefficient in the HNSCPAN index, has not yet been validated in cancer, we prioritized DSCAM for further validation and analysis. Using qRT-PCR, we observed that DSCAM mRNA was significantly overexpressed in HNSCC cell lines (CAL-27 and HN30) compared to 16HBE cells (*p* < 0.05, Fig 6A). Knockdown of DSCAM in HNSCC cells was successfully achieved (Fig 6B, C). CCK-8 assay results revealed that the DSCAM inhibition reduced the proliferative capacity of HNSCC cells (*p* < 0.05, Fig 6D). Furthermore, transwell assays and wound healing indicated that DSCAM knockdown significantly suppressed HNSCC cell and invasion and migration (*p* < 0.05, Fig 6E-G), indicating that DSCAM promotes HNSCC progression.

DSCAM knockdown in CAL27 and HN30 cells led to a marked suppression of PI3K/AKT pathway activity (Fig 6H). To further validate the mechanism, we performed rescue experiments using the PI3K activator 740Y‑P and the AKT activator SC79. The results showed that DSCAM knockdown markedly reduced the phosphorylation levels of PI3K and AKT in CAL-27 and HN30 cells. On this basis, treatment with 740Y‑P (50 μg/ml, 2 h) partially restored p‑PI3K and p‑AKT activity (Fig 6I), while treatment with SC79 (10 μM, 2 h) partially rescued p‑AKT activity (Fig 6J). These findings indicate that DSCAM participates in the regulation of PI3K/AKT signaling activity. Recent studies have demonstrated that LPS and ATP stimulation can simultaneously activate multiple programmed cell death pathways. For example, it has been reported that LPS and ATP treatment following LPCAT1 knockdown induces concurrent activation of pyroptosis, apoptosis, and necroptosis [16]. In addition, Sharma et al. showed that LPS and ATP stimulation promotes the assembly of the PANoptosome complex, and that genetic deletion of key PANoptosis components or combined treatment with a pan-caspase inhibitor and an MLKL inhibitor markedly suppresses cell death [39]. To investigate whether DSCAM knockdown influences PANoptosis, we induced pyroptosis in CAL-27 cells using LPS and ATP and examined key markers of pyroptosis. The results indicated that DSCAM knockdown promoted CASP1 cleavage and the cleavage of GSDMD and GSDME (Fig 6K). We further assessed key markers of apoptosis, and the results showed that DSCAM knockdown promoted apoptosis, as evidenced by increased cleavage fragments of CASP8 (p18) and CASP3 (p17) (Fig 6L). Finally, we examined the effect of DSCAM knockdown on necroptosis and found that it upregulated MLKL phosphorylation, indicating the induction of necroptosis (Fig 6M). These findings suggest that DSCAM knockdown promotes PANoptosis. To further clarify whether DSCAM regulates PANoptosis through the PI3K/AKT pathway, we performed rescue experiments using the AKT activator SC79. The results showed that in CAL27 cells with DSCAM knockdown, pretreatment with SC79 (10 μM, 2 h) markedly attenuated the activation of pyroptosis (GSDMD/GSDME cleavage, CASP1 activation), apoptosis (CASP3/8 cleavage), and necroptosis (MLKL phosphorylation) (Fig 6N–P). These findings suggest that the PANoptosis induced by DSCAM knockdown is at least partially mediated through the PI3K/AKT signaling pathway.

### DSCAM knockout promotes CD8^+^ T cell infiltration and antitumor activity in vivo

**Fig 7:**
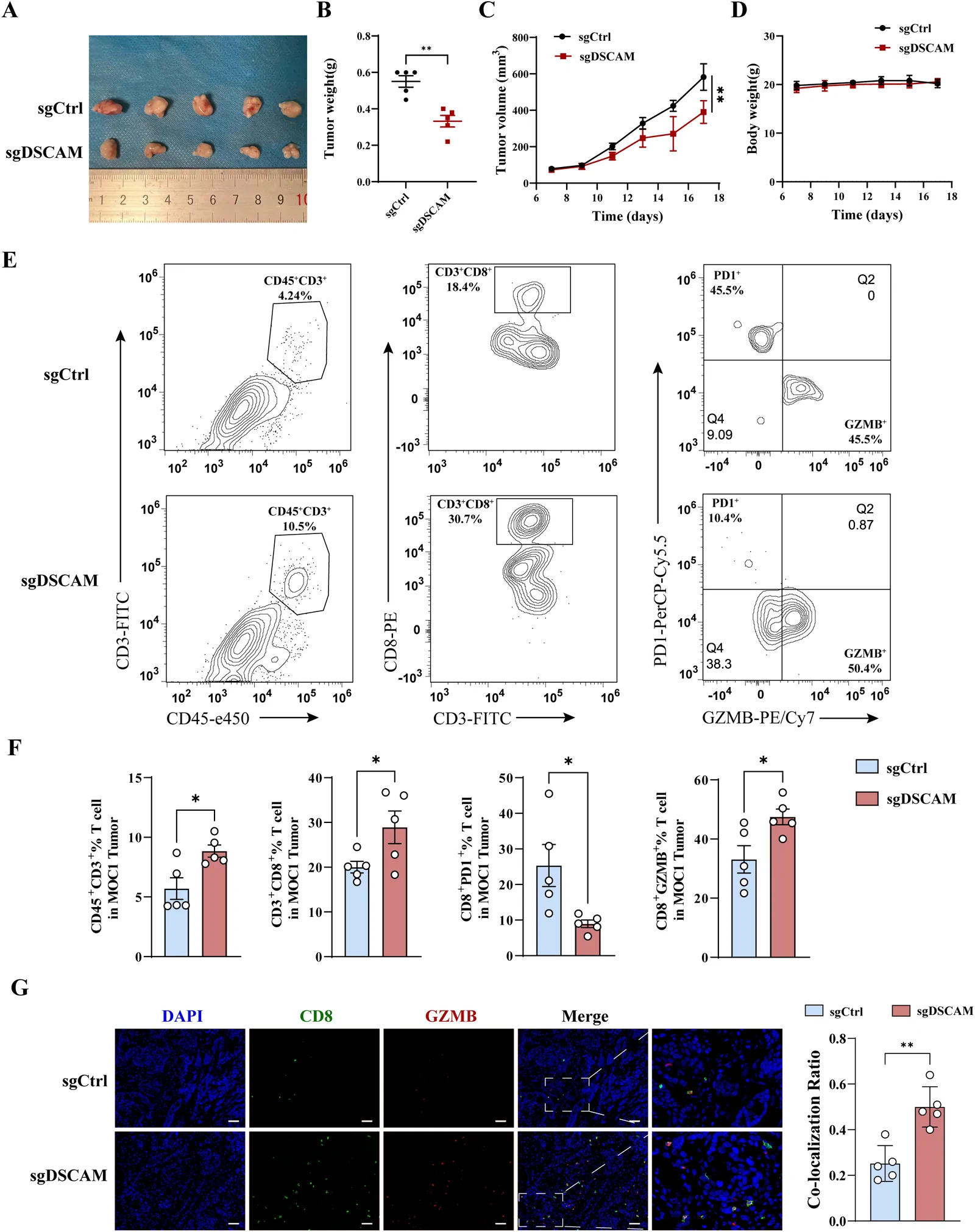
DSCAM knockout promotes CD8^+^ T-cell infiltration and antitumor activity in vivo. (A) Representative images of subcutaneous tumors surgically excised from mice.(B) Tumor weights measured at the experimental endpoint.(C) Tumor growth curves showing longitudinal monitoring of tumor volume.(D) Body weight curves of mice during the experimental period.(E, F) Flow cytometry analysis of tumor-infiltrating lymphocytes, showing the proportion of CD3⁺ and CD8^+^ T cells among CD45^+^ cells, as well as the percentage of CD8^+^ T cells expressing GZMB or PD-1. (G) Immunofluorescence staining of CD8 and Granzyme B. Images represent a selected field from a specific region of mouse tumor tissues. Scale bar: 50 µm. Data are expressed as mean ± SD, n = 5 mice per group. *p < 0.05, **p < 0.01.

To further validate the functional role of DSCAM in vivo, we generated DSCAM-knockout MOC1 cell lines using CRISPR-Cas9 technology (S2 Fig, provided in the raw images) and implanted either wild-type or DSCAM-deficient MOC1 cells subcutaneously into C57BL/6 mice. Tumor growth was monitored over time, and we observed a pronounced suppression of tumor progression in the DSCAM-deficient group compared with the wild-type control. This growth inhibition was consistent across both tumor volume and tumor weight measurements, highlighting the essential role of DSCAM in sustaining tumor development (Fig 7A–D). To explore whether the observed tumor suppression was associated with altered immune responses, we performed flow cytometric analyses of tumor-infiltrating lymphocytes. Remarkably, DSCAM deficiency led to a robust increase in CD8^+^ T cell infiltration within the tumor microenvironment. In addition, functional characterization of these CD8^+^ T cells revealed elevated expression of granzyme B (GZMB), a key cytotoxic effector molecule, and a concomitant reduction in PD-1 expression, suggesting enhanced cytotoxic activity and reduced exhaustion status (Fig 7E, F). We further performed immunofluorescence staining on tumor tissues. The results showed that, compared with the control group, intratumoral CD8^+^ T cell infiltration was significantly increased in the DSCAM-deficient group. In addition, co-staining for CD8 and GZMB revealed a marked elevation in the number of CD8^+^ GZMB^+^ effector T cells in DSCAM-deficient tumors (Fig 7G).

## Discussion

Although immune checkpoint inhibitors (ICIs) have transformed the therapeutic landscape of advanced HNSCC, durable clinical responses remain limited to a subset of patients, and resistance remains a major challenge [40–42]. The complex heterogeneity of HNSCC, immunosuppressive tumor microenvironment (TME), limited availability of reliable biomarkers, and resistance to conventional therapies collectively hinder optimal treatment efficacy [2,43]. PANoptosis has recently attracted increasing attention due to its potential roles in tumor regulation and immune modulation [6,44]. Although certain molecular mechanisms related to PANoptosis in other types of cancers have been studied, research on its role in HNSCC remains relatively limited. In this study, we identified three distinct PANoptosis molecular subtypes with different prognostic and immune characteristics and developed the HNSCPAN-index to evaluate prognosis, drug sensitivity, and immunotherapy response. Furthermore, functional validation revealed DSCAM as a potential regulator of PANoptosis resistance and immune remodeling in HNSCC. Collectively, our findings provide new insights into the role of PANoptosis in HNSCC and highlight potential biomarkers and therapeutic vulnerabilities for future investigation.

We investigated the immune characteristics associated with distinct PANoptosis subtypes in HNSCC. Our analysis revealed that PANoptosis Cluster C exhibited a markedly higher level of CD8^+^ T cell infiltration in the TME compared to that of Clusters A and B. Furthermore, Cluster C exhibited greater enrichment in antigen processing and presentation pathways, consistent with previous findings that HNSCC tumors with robust CD8^+^ T cell infiltration are associated with improved prognoses, likely due to enhanced effector cell function and increased tumor cell killing [2]. The expression profiles of these T cells may also predict the responses to checkpoint immunotherapy [45]. Single-cell sequencing of HNSCC further revealed distinct populations of cytotoxic CD8^+^ T cells, including CD8^+^ T and CD8^+^ T exhausted cells, with differential expression of co-inhibitory receptors such as PD1 and CTLA4, along with other genes linked to T cell dysfunction. This suggests that immune checkpoint inhibitors (e.g., PD-1/PD-L1 or CTLA-4 inhibitors) may relieve T cell inhibition and enhance tumor cell killing in this subset of patients [29]. This implies that modulation of these pathways in populations with high HNSCPAN-index scores may produce significant enhancement of HNSCC immunotherapy.

To enhance prognostic prediction for patients with HNSCC, we identified DEGs across the three PANoptosis clusters that were significantly associated with prognosis. Based on these DEGs, we constructed an HNSCPAN-index that was validated for predictive accuracy, independence, and clinical applicability. Additionally, we developed a prognostic nomogram by integrating factors such as sex, tumor stage and age, thereby enabling individualized survival probability prediction. Consequently, the HNSCPAN-index emerged as a reliable and effective tool for predicting the HNSCC prognosis. We further evaluated its ability to predict the immune landscape and therapeutic response in patients with HNSCC. Patients classified as low-risk exhibited higher levels of M1 macrophages and CD8^+^ T cells, correlating with better prognoses than that of those in the high-risk group, who possessed elevated levels of M0 macrophages and resting CD4^+^ memory T cells, both of which contributed to immune suppression and tumor progression [46,47]. These findings highlight the critical role of macrophages in HNSCC prognosis and suggest their potential as macrophage-targeted therapies. Notably, CD8^+^ T cell and macrophage interactions in the HNSCC TME were characterized by predicted HAVCR2 (TIM-3) -LGALS9, CD274 (PD-L1)-PDCD1(PD-1) and TIGIT-NECTIN2 interactions that are likely key to tumor rejection [28]. This implies that modulation of these pathways in populations with high HNSCPAN-index scores may produce significant enhancement of HNSCC immunotherapy. Previous studies have demonstrated co-localization of T cells with PD-L1^+^ macrophages in inflammatory HNSCC lesions [48]. This co-localization suggests that these cells may be jointly involved in the immune escape mechanism of tumor suppression through the immune checkpoint pathway. Therefore, as new ICR-targeting therapies are approved for the treatment of various malignancies, a better understanding of the primary cellular sources and expression patterns of ICR ligands in the HNSCC TME is of paramount importance [49]. Our findings suggest that low-risk patients, characterized by a high expression of immune checkpoint genes, may respond favorably to ICI therapy, as corroborated by the TIDE scores, IMvigor210 cohort data, and TMB analysis. Notably, patients with higher TMB, which is often associated with improved outcomes, were enriched in the low-risk group that also exhibited lower TIDE scores [50]. Furthermore, in patients with HNSCC receiving immunotherapy, the incidence of CR/PR was higher in the low-risk group, demonstrating that a lower risk score was closely associated with better clinical outcomes and greater responsiveness to immunotherapy.

Among the genes with the highest mutation frequencies, TP53 showed the most significant intergroup difference, with a mutation rate of 75% in the high-risk group. The enrichment of TP53 mutations in the high-risk group is particularly meaningful in light of emerging evidence regarding p53-regulated PANoptosis. A recent review by Liu et al. points out that in addition to its classical role in regulating apoptosis, p53 also regulates multiple non-apoptotic cell death pathways, including PANoptosis [51].Yu et al. found that TP53 mutations drive the upregulation of RIPK4 expression in colorectal cancer, thereby promoting resistance to PANoptosis and facilitating metastasis [52]. TP53 mutations in high-risk HNSCC tumors may promote tumor progression and poor prognosis by interfering with the normal regulation of PANoptosis.

Given the potential impact of PANoptosis on HNSCC heterogeneity and associated clinical outcomes, we developed a prognostic model based on six PANoptosis-related DEGs to quantify the HNSCPAN-index. Within this framework, DSCAM emerged as a previously uncharacterized oncogenic driver. We demonstrated that DSCAM is aberrantly overexpressed in HNSCC and that it’s silencing profoundly suppresses tumor cell proliferation and migration, consistent with its adverse prognostic impact. Mechanistically, previous studies have shown that inhibition of the DSCAM/PAK1 axis can reverse neurogenesis defects in induced pluripotent stem cells [53]. In parallel, suppression of the PI3K/AKT pathway has been reported to modulate PAK1 signaling and enhance antitumor immune responses in lung cancer [54]. Moreover, accumulating evidence has identified the PI3K/AKT pathway as a central regulatory hub of PANoptosis [55,56]. Based on these findings, we hypothesized that DSCAM may regulate cell death in HNSCC through the PI3K/AKT signaling cascade. Our results further confirmed that DSCAM indeed regulates PANoptosis via the PI3K/AKT pathway. Moreover, DSCAM deficiency in vivo not only curtailed tumor growth but also enhanced immune surveillance, as evidenced by increased CD8^+^ T cell infiltration and augmented cytotoxic function with reduced exhaustion phenotypes.

This study has several limitations. Although the HNSCPAN-index showed prognostic and therapeutic predictive value across multiple datasets, further validation in large, independent, prospective HNSCC cohorts is required, as the IMvigor210 cohort was not HNSCC-specific. Future studies should also compare the HNSCPAN-index with established prognostic signatures and incorporate multi-omics approaches to better characterize PANoptosis and DSCAM-mediated regulation in the tumor microenvironment. Moreover, DSCAM overexpression models are needed to further clarify its upstream regulatory mechanisms.

## Conclusion

In summary, we identified three molecular subtypes of PANoptosis in HNSCC and developed the HNSCPAN-index as a robust predictor of prognosis and therapeutic response. Functional validation revealed DSCAM as a novel oncogenic driver that sustains PI3K/AKT signaling, suppresses PANoptosis, and facilitates immune evasion, while its loss reactivated cell death and enhanced CD8 ⁺ T cell activity. These findings highlight the HNSCPAN-index and DSCAM as promising tools for precision prognosis and immunotherapy in HNSCC.

## Supporting information

S1 Fig Correlation between promoter methylation and mRNA expression of HNSCPAN-index genes in TCGA-HNSC. Panels (**A**–**F**) show the correlations for DSCAM (**A**), NT5E (**B**), MIAT (**C**), CXCL1 (**D**), IL12RB2 (**E**), and AKR1C3 (**F**).
(DOCX)

S2 Fig Raw images. (PDF)

S1 File Includes the following three datasets: TCGA-HNSC-count (gene expression counts), TCGA-HNSC-Survival (survival data), and TCGA-HNSC-Clinical (clinical characteristics). (ZIP)

S2 File Contains the geoClinic and series_matrix datasets from GSE65858. (ZIP)

S1 Table PANoptosis gene list. (DOCX)

S2 Table Primer sequence of RT-qPCR. (DOCX)

S3 Table Western blot antibody information. (DOCX)

S4 Table Flow cytometry antibody information. (DOCX)
