# Supplementary material for: PANoptosis-based HNSCPAN-index predicts prognosis and reveals DSCAM as a therapeutic target in head and neck squamous cell carcinoma
Source: PLoS One. 2026 Sep 15;21(9):e0344957. doi: 10.1371/journal.pone.0344957 (PMC13577361; doi:10.1371/journal.pone.0344957)
Supplement: Supplementary file 1 [file pone.0344957.s002.pdf]

Fig 6

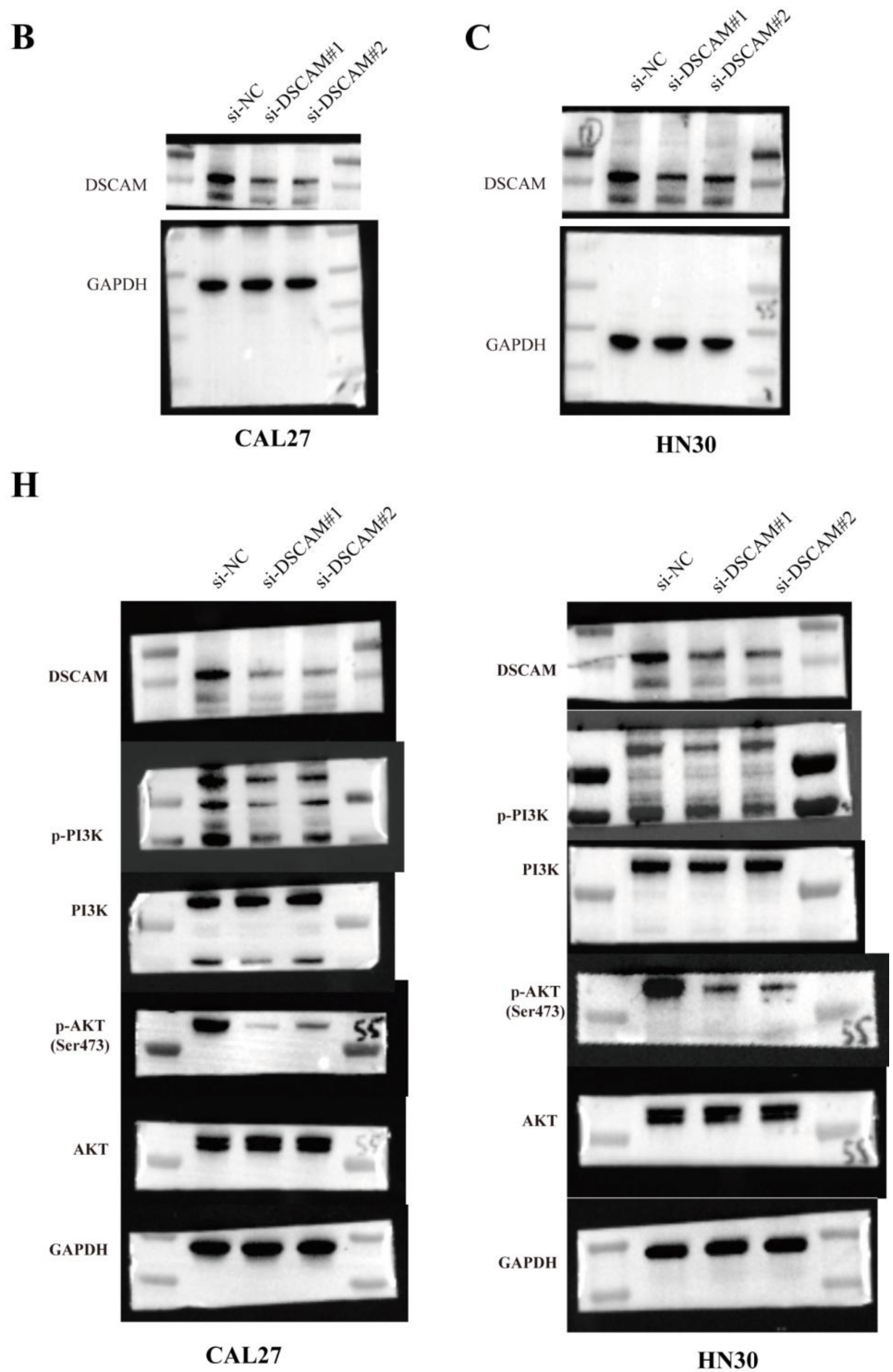

B
si-NC
si-DSCAM#1
si-DSCAM#2
DSCAM
GAPDH
CAL27
C
si-NC
si-DSCAM#1
si-DSCAM#2
DSCAM
GAPDH
HN30
H
si-NC
si-DSCAM#1
si-DSCAM#2
DSCAM
p-PI3K
PI3K
p-AKT (Ser473)
AKT
GAPDH
CAL27
si-NC
si-DSCAM#1
si-DSCAM#2
DSCAM
p-PI3K
PI3K
p-AKT (Ser473)
AKT
GAPDH
HN30

Fig 6

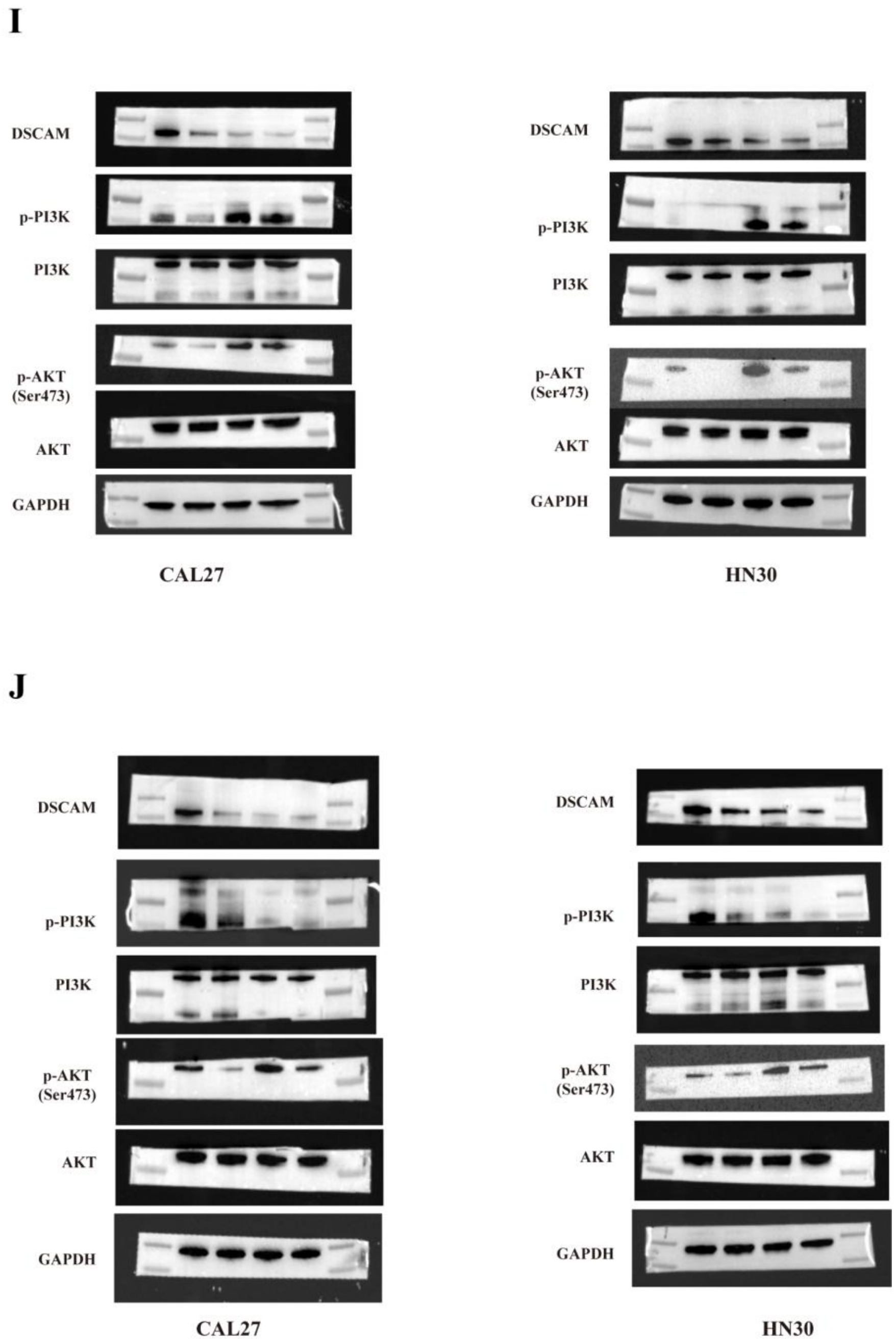
I
DSCAM
p-PI3K
PI3K
p-AKT
(Ser473)
AKT
GAPDH
CAL27
DSCAM
p-PI3K
PI3K
p-AKT
(Ser473)
AKT
GAPDH
HN30
J
DSCAM
p-PI3K
PI3K
p-AKT
(Ser473)
AKT
GAPDH
CAL27
DSCAM
p-PI3K
PI3K
p-AKT
(Ser473)
AKT
GAPDH
HN30

Fig 6

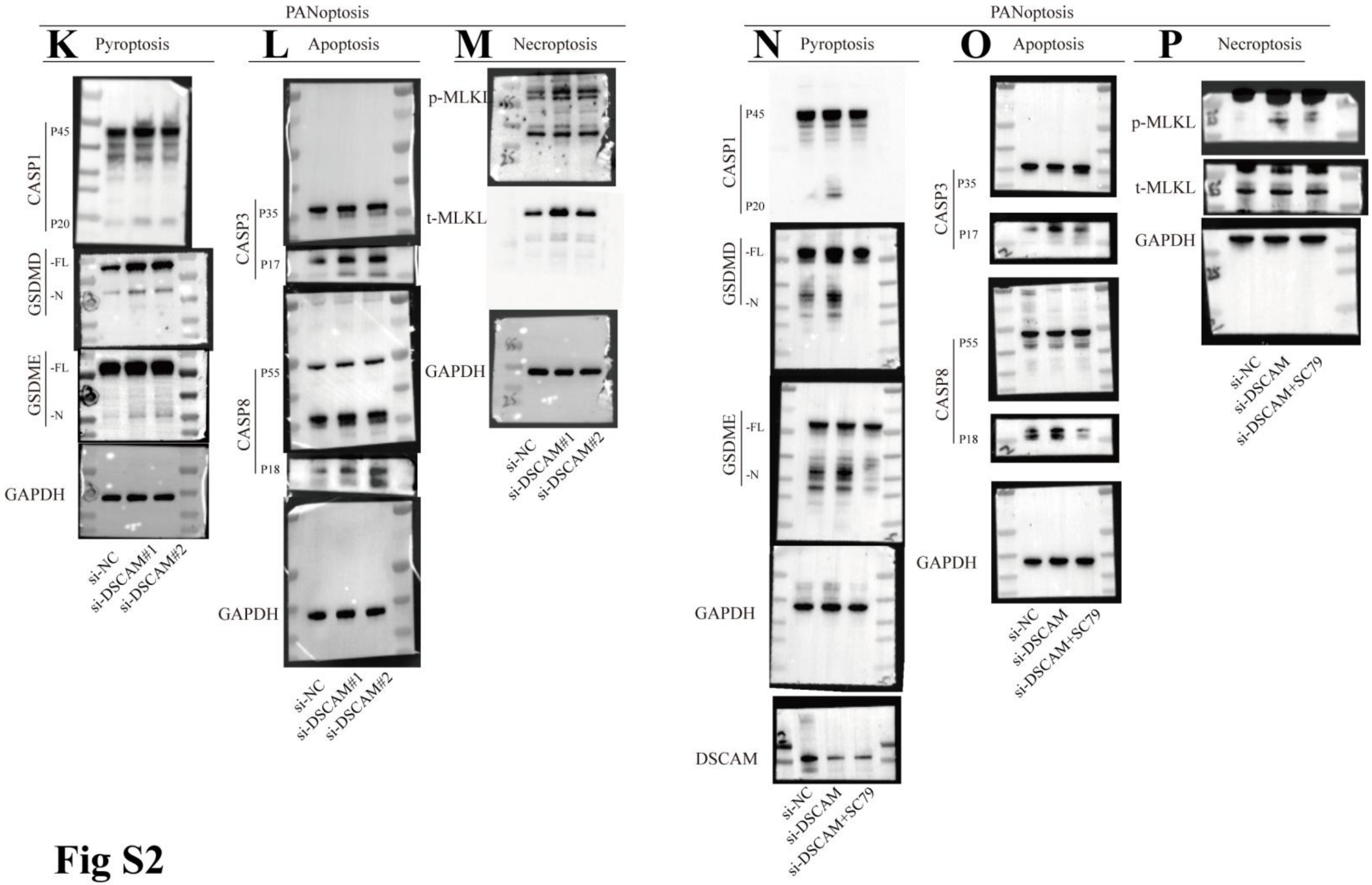
PANoptosis
K Pyroptosis
L Apoptosis
M Necroptosis
CASP1
P45
P20
GSDMD
-FL
-N
GSDME
-FL
-N
GAPDH
si-NC
si-DSCAM#1
si-DSCAM#2
CASP3
P35
P17
CASP8
P55
P18
GAPDH
si-NC
si-DSCAM#1
si-DSCAM#2
p-MLKL
t-MLKL
GAPDH
si-NC
si-DSCAM#1
si-DSCAM#2
PANoptosis
N Pyroptosis
O Apoptosis
P Necroptosis
CASP1
P45
P20
GSDMD
-FL
-N
GSDME
-FL
-N
GAPDH
DSCAM
si-NC
si-DSCAM
si-DSCAM+SC79
CASP3
P35
P17
CASP8
P55
P18
GAPDH
si-NC
si-DSCAM
si-DSCAM+SC79
p-MLKL
t-MLKL
GAPDH
si-NC
si-DSCAM
si-DSCAM+SC79

Fig S2

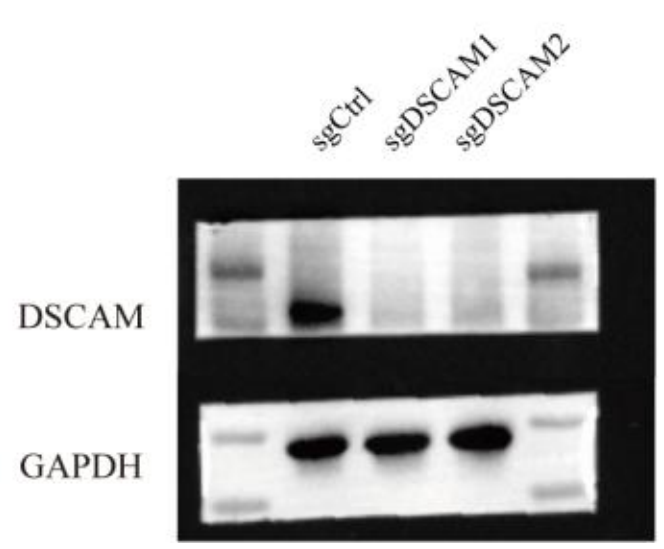
sgCtrl
sgDSCAM1
sgDSCAM2
DSCAM
GAPDH
