# Supplementary material for: PANoptosis-based HNSCPAN-index predicts prognosis and reveals DSCAM as a therapeutic target in head and neck squamous cell carcinoma
Source: PLoS One. 2026 Sep 15;21(9):e0344957. doi: 10.1371/journal.pone.0344957 (PMC13577361; doi:10.1371/journal.pone.0344957)
Supplement: Supplementary file 2 [file pone.0344957.s001.docx]

**Supplementary Figure S1. Correlation between promoter methylation and mRNA expression of HNSCPAN-index genes in TCGA-HNSC. Panels (A–F) show the correlations for DSCAM (A), NT5E (B), MIAT (C), CXCL1 (D), IL12RB2 (E), and AKR1C3 (F).**
