# Supplementary material for: PANoptosis-based HNSCPAN-index predicts prognosis and reveals DSCAM as a therapeutic target in head and neck squamous cell carcinoma
Source: PLoS One. 2026 Sep 15;21(9):e0344957. doi: 10.1371/journal.pone.0344957 (PMC13577361; doi:10.1371/journal.pone.0344957)
Supplement: Supplementary file 3 [file pone.0344957.s005.docx]

**S1 Table. PANoptosis gene list.**

| Gene | Type |
| --- | --- |
| AIFM1 | Apoptosis |
| AKT3 | Apoptosis |
| APAF1 | Apoptosis |
| APPL1 | Apoptosis |
| BMF | Apoptosis |
| BNIP3L | Apoptosis |
| BOK | Apoptosis |
| CASP10 | Apoptosis |
| CASP2 | Apoptosis |
| CASP6 | Apoptosis |
| CASP7 | Apoptosis |
| CASP8 | Apoptosis |
| CD14 | Apoptosis |
| CHUK | Apoptosis |
| CRADD | Apoptosis |
| DFFA | Apoptosis |
| DFFB | Apoptosis |
| E2F1 | Apoptosis |
| HMGB2 | Apoptosis |
| IGF1 | Apoptosis |
| LY96 | Apoptosis |
| PPP3R1 | Apoptosis |
| TFDP1 | Apoptosis |
| TICAM1 | Apoptosis |
| TNFRSF1A | Apoptosis |
| TNFSF10 | Apoptosis |
| TP73 | Apoptosis |
| TRAF2 | Apoptosis |
| UACA | Apoptosis |
| UNC5B | Apoptosis |
| YWHAE | Apoptosis |
| FADD | Necroptosis |
| FAS | Necroptosis |
| FASLG | Necroptosis |
| MLKL | Necroptosis |
| RIPK1 | Necroptosis |
| RIPK3 | Necroptosis |
| TLR3 | Necroptosis |
| TNF | Necroptosis |
| BAK1 | Necroptosis |
| BAX | Pyroptosis |
| CASP1 | Pyroptosis |
| CASP3 | Pyroptosis |
| CASP4 | Pyroptosis |
| CASP5 | Pyroptosis |
| CHMP2A | Pyroptosis |
| CHMP2B | Pyroptosis |
| CHMP3 | Pyroptosis |
| CHMP4A | Pyroptosis |
| CHMP4B | Pyroptosis |
| CHMP4C | Pyroptosis |
| CHMP6 | Pyroptosis |
| CHMP7 | Pyroptosis |
| CYCS | Pyroptosis |
| ELANE | Pyroptosis |
| GSDMD | Pyroptosis |
| GSDME | Pyroptosis |
| GZMB | Pyroptosis |
| HMGB1 | Pyroptosis |
| IL18 | Pyroptosis |
| IL1A | Pyroptosis |
| IL1B | Pyroptosis |
| IRF1 | Pyroptosis |
| IRF2 | Pyroptosis |
