# Supplementary material for: PANoptosis-based HNSCPAN-index predicts prognosis and reveals DSCAM as a therapeutic target in head and neck squamous cell carcinoma
Source: PLoS One. 2026 Sep 15;21(9):e0344957. doi: 10.1371/journal.pone.0344957 (PMC13577361; doi:10.1371/journal.pone.0344957)
Supplement: Supplementary file 4 [file pone.0344957.s006.docx]

**S2 Table. Primer sequence of RT-qPCR.**

| Genes | Sequences (5’-3’) |
| --- | --- |
| DSCAM (human) | F: AGAGCTTCGCGAATGTTTTCA |
|  | R: GGCAAACACTACCTCTTGCAG |
| GAPDH (human) | F: ACAGCCTCAAGATCATCAGC |
|  | R: GGTCATGAGTCCTTCCACGAT |

Note: F, forward; R, reverse.
