# Supplementary material for: PANoptosis-based HNSCPAN-index predicts prognosis and reveals DSCAM as a therapeutic target in head and neck squamous cell carcinoma
Source: PLoS One. 2026 Sep 15;21(9):e0344957. doi: 10.1371/journal.pone.0344957 (PMC13577361; doi:10.1371/journal.pone.0344957)
Supplement: Supplementary file 5 [file pone.0344957.s007.docx]

**S3 Table. Western blot antibody information.**

| **Antibody** | **Manufacturer** | **CAS** | **Dilution ratio** |
| --- | --- | --- | --- |
| cleaved caspase-3 (human) | Cell Signaling, USA | #9664 | 1:1000 |
| caspase-3 (human) | Cell Signaling, USA | #9662 | 1:1000 |
| caspase-8 (human) | Cell Signaling, USA | #4790 | 1:1000 |
| cleaved caspase-8 (human) | Cell Signaling, USA | #85921 | 1:1000 |
| caspase-1 (human) | Proteintech, China | Cat No. 22915-1-AP | 1:6000 |
| GSDME (human) | Proteintech, China | Cat No. 13075-1-AP | 1:1000 |
| cleaved GSDME (human) | ABclonal, USA | A23072 | 1:1000 |
| GSDMD (human) | ABclonal, USA | A21894 | 1:1000 |
| MLKL (human) | ABclonal, USA | Ab52903 | 1:1000 |
| p-MLKL(human) | ABclonal, USA | A1244 | 1:1000 |
| Phospho-PI3 Kinase p85 | Cell Signaling, USA | #17366 | 1:1000 |
| PI3K p110 | Santa Cruz | sc-8010 | 1:1000 |
| Phospho-Akt Ser473 | Cell Signaling, USA | #4060 | 1:1000 |
| Akt | Cell Signaling, USA | #2920 | 1:1000 |
| GAPDH (human) | Proteintech, China | Cat No. 60004-1-Ig | 1:10000 |
