# Supplementary material for: PANoptosis-based HNSCPAN-index predicts prognosis and reveals DSCAM as a therapeutic target in head and neck squamous cell carcinoma
Source: PLoS One. 2026 Sep 15;21(9):e0344957. doi: 10.1371/journal.pone.0344957 (PMC13577361; doi:10.1371/journal.pone.0344957)
Supplement: Supplementary file 6 [file pone.0344957.s008.docx]

**S4 Table. Flow cytometry antibody information.**

| **Antibody** | **Manufacturer** | | | **CAS** | **Dilution ratio** |
| --- | --- | --- | --- | --- | --- |
| FITC Anti-Mouse CD3 Antibody | | Elabscience, China | E-AB-F1013C | | 1:200 |
| lab Fluor® Violet 450 Anti-Mouse CD45 Antibody | | Elabscience, China | E-AB-F1136Q | | 1:200 |
| CD8a Monoclonal Antibody(mouse) | | eBioscience™, USA | # 12-0081-82 | | 1:200 |
| Granzyme B Monoclonal Antibody(mouse) | | eBioscience™, USA | # 25-8898-82 | | 1:200 |
| PerCP/Cyanine5.5 Anti-Mouse CD279/PD-1 Antibody | | Elabscience, China | E-AB-F1131J | | 1:200 |
