# Supplementary material for: PANoptosis-based HNSCPAN-index predicts prognosis and reveals DSCAM as a therapeutic target in head and neck squamous cell carcinoma
Source: PLoS One. 2026 Sep 15;21(9):e0344957. doi: 10.1371/journal.pone.0344957 (PMC13577361; doi:10.1371/journal.pone.0344957)
Supplement: Supplementary file 7 [file pone.0344957.s003.zip › Data Source.docx]

TCGA database: <https://portal.gdc.cancer.gov/>

TCGA-HNSC-count:

<https://gdc-hub.s3.us-east-1.amazonaws.com/download/TCGA-HNSC.star_counts.tsv.gz>

TCGA-HNSC-Survival:

<https://gdc-hub.s3.us-east-1.amazonaws.com/download/TCGA-HNSC.survival.tsv.gz>

TCGA-HNSC-Clinical:

<https://portal.gdc.cancer.gov>

GSE145281 (IMvigor210 immunotherapy cohort):

<https://www.ncbi.nlm.nih.gov/geo/query/acc.cgi?acc=GSE145281>

<http://research-pub.gene.com/IMvigor210CoreBiologies/packageVersions/IMvigor210CoreBiologies_1.0.0.tar.gz>
